# An Intelligent Ship Detection Algorithm Based on Visual Sensor Signal Processing for AIoT-Enabled Maritime Surveillance Automation

**DOI:** 10.3390/s26030767

**Published:** 2026-01-23

**Authors:** Liang Zhang, Yueqiu Jiang, Wei Yang, Bo Liu

**Affiliations:** 1 Graduate School, Shenyang Ligong University, Shenyang 110159, China; syzhangliang@163.com (L.Z.); liubosylg@163.com (B.L.); 2 School of Information Science and Engineering, Shenyang Ligong University, Shenyang 110159, China; 3 Faculty of Equipment Engineering, Shenyang Ligong University, Shenyang 110159, China; weiyang@sylu.edu.cn

**Keywords:** artificial intelligence of things, ship detection, automated maritime surveillance, signal processing, oriented bounding box, sensor data analysis

## Abstract

Oriented object detection constitutes a fundamental yet challenging task in Artificial Intelligence of Things (AIoT)-enabled maritime surveillance, where real-time processing of dense visual streams is imperative. However, existing detectors suffer from three critical limitations: sequential attention mechanisms that fail to capture coupled spatial–channel dependencies, unconstrained deformable convolutions that yield unstable predictions for elongated vessels, and center-based distance metrics that ignore angular alignment in sample assignment. To address these challenges, we propose JAOSD (Joint Attention-based Oriented Ship Detection), an anchor-free framework incorporating three novel components: (1) a joint attention module that processes spatial and channel branches in parallel with coupled fusion, (2) an adaptive geometric convolution with two-stage offset refinement and spatial consistency regularization, and (3) an orientation-aware Adaptive Sample Selection strategy based on corner-aware distance metrics. Extensive experiments on three benchmarks demonstrate that JAOSD achieves state-of-the-art performance—94.74% mAP on HRSC2016, 92.43% AP_50_ on FGSD2021, and 80.44% mAP on DOTA v1.0—while maintaining real-time inference at 42.6 FPS. Cross-domain evaluation on the Singapore Maritime Dataset further confirms robust generalization capability from aerial to shore-based surveillance scenarios without domain adaptation.

## 1. Introduction

**Figure 1:**
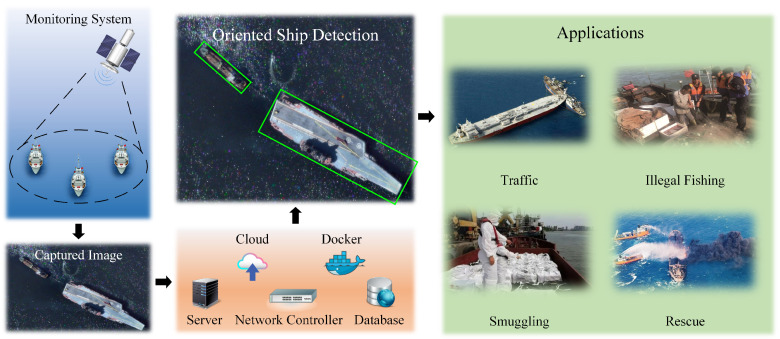
AIoT-enabled maritime surveillance workflow. Heterogeneous cameras and satellites stream imagery to edge processors running JAOSD; the resulting oriented detections support traffic management, anomaly alerting, and rescue coordination.

The proliferation of Artificial Intelligence of Things (AIoT) has transformed maritime surveillance through intelligent integration of heterogeneous sensor networks for vessel traffic management, collision avoidance, and safety monitoring [1,2,3], as illustrated in Figure 1. Central to these systems is the capability to detect ships with arbitrary orientations under challenging conditions, including severe illumination variations, wake-induced clutter, and stringent real-time processing constraints [4]. Conventional horizontal bounding box detectors are inherently limited when handling overlapping vessels and objects with extreme aspect ratios, motivating the development of oriented object detection methods that employ rotated anchors, deformable convolutions, and adaptive assignment strategies [5,6,7]. Despite these advances, three fundamental challenges remain inadequately addressed: (1) sequential spatial–channel attention mechanisms [8] fail to capture the coupled dependencies essential for discriminating densely berthed vessels; (2) unconstrained deformable kernels yield unstable orientation predictions for elongated structures [9]; and (3) fixed IoU thresholds exhibit poor generalization to anisotropic maritime environments [10,11].

To address these limitations, we propose JAOSD (Joint Attention-based Oriented Ship Detection), an anchor-free detection framework that integrates multi-scale joint attention, adaptive geometric convolution (AGC), and maritime-specific Adaptive Sample Selection (ASS). The main contributions are summarized as follows:

(1) We introduce a joint attention module (JAM) that leverages parallel spatial–channel branches with efficient pooling-based architecture, achieving coupled feature recalibration addressing the limitation of sequential attention mechanisms that fail to capture coupled dependencies.
(2) We introduce an adaptive geometric convolution (AGC) mechanism incorporating two-stage offset refinement and spatial consistency regularization, enabling precise alignment of sampling points with vessel structures while mitigating angle-periodicity ambiguities.
(3) We introduce an Adaptive Sample Selection (ASS) strategy grounded in statistical analysis of oriented distances, incorporating μ+σ threshold rules with center-inclusion constraints to accommodate extreme aspect ratios and dense berthing scenarios.
(4) Experiments across three benchmark datasets establish state-of-the-art performance with statistical significance testing (p<0.001). Cross-domain evaluation on the Singapore Maritime Dataset and edge deployment on Jetson Orin NX confirm practical applicability for AIoT maritime surveillance.

The remainder of this paper is organized as follows. Section 2 surveys the AIoT maritime surveillance, oriented detection, and adaptive assignment literature. Section 3 formalizes the notation and details JAOSD’s JAM, AGC, and ASS components. Section 4 presents datasets, implementation details, ablation studies, SOTA comparisons, and edge deployment evaluation. Section 5 concludes with contributions and future directions.

## 2. Related Works

This section reviews key advances in AIoT maritime surveillance, oriented object detection methodologies, and ship detection techniques in remote sensing imagery.

### 2.1. AIoT-Enabled Maritime Applications

AIoT has fundamentally transformed maritime surveillance through the integration of sensor networks and artificial intelligence for trajectory prediction and anomaly detection [12,13,14,15,16,17,18,19]. Key enabling technologies include AIS-based vessel security systems [20], adaptive multi-source data fusion for trajectory prediction [21,22], and maritime IoT communication architectures that address coverage, latency, and reliability challenges [23,24,25]. Recent advances during 2024–2025 include distributed blockchain-based traffic supervision [26], edge computing paradigms for real-time ship detection [27], and federated learning frameworks for privacy-preserving multi-source detection [28]. These innovations collectively establish ship detection as the foundational capability for AIoT-enabled maritime surveillance systems [29,30,31,32].

### 2.2. Oriented Object Detection

Oriented object detection (OOD) overcomes the limitations of axis-aligned bounding boxes to accommodate arbitrary orientations prevalent in remote sensing imagery. Foundational single-stage detectors such as SSD [33] established efficient detection paradigms that continue to influence contemporary architectures. Subsequent advances include ORCNN-X’s multi-scale fusion mechanism [34], WFR’s adaptive refinement strategy [35], RoI-Transformer’s explicit rotation handling [36], high-quality angle prediction methodologies [37], and Rotated-IoU loss formulations [38]. Recent YOLO variants spanning v5–v8 [39,40,41,42], v10 [43], and the lightweight v11 optimized for ship detection [44], along with extensions such as YOLOX [45], remain constrained by fixed convolution kernels that inadequately address maritime scenarios. Task-aligned methods exemplified by TOOD [46] demonstrate improved classification–localization alignment but lack the geometric adaptability requisite for extreme ship aspect ratios.

Representative baseline methods comprise RetinaNet-O [47], FCOS-O [48], RoI Transformer [49], Gliding Vertex [50], R^3^Det [51], S^2^A-Net [52], ReDet [53], Oriented R-CNN [54], and Oriented RepPoints [7]. Nevertheless, maritime-specific challenges persist, including extreme aspect ratios approaching 15:1, dense berthing configurations with IoU exceeding 0.8, and substantial scale variations.

Attention mechanisms have proven effective for feature enhancement in detection tasks. SE-Net [55] introduces channel-wise attention through squeeze-and-excitation operations, while CBAM [8] extends this paradigm with sequential spatial attention. However, these serial processing pipelines are inherently incapable of capturing the coupled spatial–channel dependencies critical for maritime scenarios. Dual attention networks [56] achieve parallel position–channel attention through self-attention mechanisms but incur prohibitive $O(N^{2})$ complexity unsuitable for real-time detection; efficient channel attention [57] reduces parametric overhead but remains confined to channel-only processing. Similarly, deformable convolutions [58,59] enable adaptive receptive fields but lack the explicit geometric constraints essential for elongated ship structures. Our proposed JAM addresses these limitations through lightweight parallel spatial–channel processing with pooling-based design (less than 5% overhead), while AGC incorporates two-stage refinement with spatial consistency regularization specifically tailored for maritime objects.

Recent advances in dual-space and non-Euclidean representation learning offer complementary perspectives for visual understanding. Dual-space methods have demonstrated effectiveness in video anomaly detection [60,61,62] and person re-identification [63]. For aerial imagery, Pareto refocusing addresses scale imbalance in drone-view detection [64], while A2Seek provides reasoning-centric benchmarks for aerial anomaly understanding [65]. Although our current approach operates within the Euclidean feature space with geometric adaptations, these dual-space representations constitute promising future directions for maritime anomaly detection beyond conventional object detection paradigms.

### 2.3. Ship Detection in Remote Sensing Images

Ship detection in remote sensing imagery has evolved through three distinct paradigms. Traditional methodologies employed sliding-window classifiers with handcrafted features, achieving limited robustness to scale variations. The advent of convolutional neural networks introduced end-to-end frameworks: RoI-based methods [49] excel at precise localization but suffer from anchor design complexity, whereas anchor-free approaches [66] offer architectural simplicity but struggle with the extreme aspect ratios characteristic of maritime vessels.

Multi-modal approaches leverage synthetic aperture radar (SAR) imagery for all-weather oriented detection [67], while optical methods employ scene complexity analysis for real-time processing [68]. Recent advances address specific maritime challenges through diverse strategies: lightweight architectures [69,70] target edge deployment scenarios; saliency-guided sampling [71] enhances discriminative feature selection; deformable convolutions [72,73] enable adaptive receptive field modeling; attention mechanisms [74,75] improve feature discrimination capability; and weather-robust features [76,77] ensure operational reliability under adverse conditions. Extended YOLO frameworks including YOLOv8-OBB [41] and lightweight variants [44] have demonstrated competitive efficiency on general oriented bounding box benchmarks.

Despite these advances, existing approaches share fundamental limitations when applied to maritime surveillance: (1) sequential attention mechanisms fail to capture the coupled spatial–channel dependencies critical for dense berthing scenarios where vessels exhibit IoU exceeding 0.8; (2) standard deformable convolutions lack explicit geometric constraints for elongated ship structures with aspect ratios reaching 15:1; and (3) center-based Euclidean distance metrics in sample assignment [10] presuppose isotropic objects, thereby ignoring the angular alignment crucial for oriented ship detection. These identified gaps motivate our JAOSD framework, which incorporates parallel joint attention, geometry-constrained adaptive convolution, and orientation-aware sample selection.

## 3. Proposed Method

**Figure 2:**
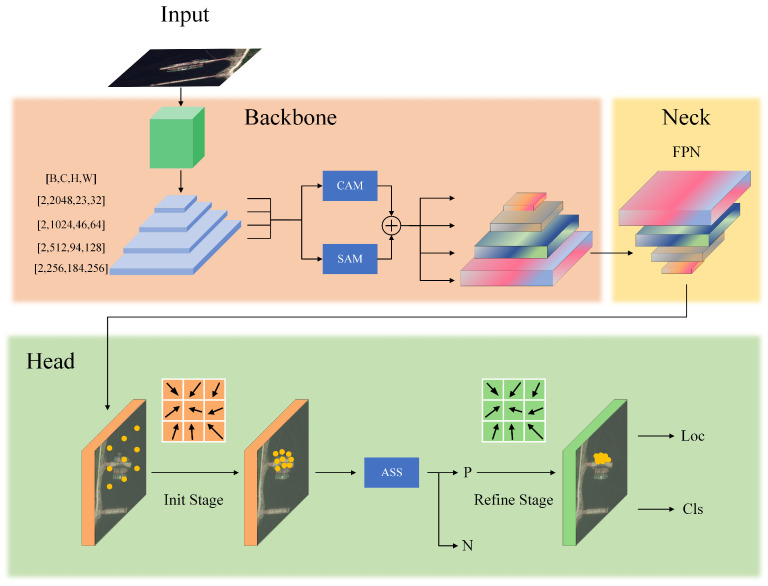
Overall framework of the proposed Joint Attention-based Oriented Ship Detection (JAOSD) method. The network architecture integrates a ResNet101 backbone enhanced by parallel joint attention modules (CAM and SAM), a Feature Pyramid Network (FPN) neck for multi-scale feature fusion, and a two-stage detection head with an Adaptive Sample Selection (ASS) strategy for oriented bounding box prediction.

This section presents the proposed JAOSD framework in detail. To extract discriminative ship features from complex maritime backgrounds while suppressing redundant information, we introduce a multi-scale spatial–channel joint attention module. In contrast to conventional direction regression methods, we employ a more refined and flexible orientation representation approach through adaptive geometric convolution (AGC), which generates adaptive sampling point sets. AGC enables sampling points to migrate toward localized, high-density, and semantically informative regions based on input features, thereby better accommodating ships with arbitrary orientations and diverse morphologies. Unlike anchor-based methods that densely deploy anchors—resulting in excessive parameters, substantial computational overhead, and additional hyperparameter tuning—we adopt an anchor-free detection paradigm and employ the Adaptive Sample Selection (ASS) strategy to adaptively designate positive and negative samples based on the statistical properties of ship objects. Figure 2 presents an architectural overview of the proposed oriented ship detection framework.

### 3.1. Multi-Scale Spatial–Channel Joint Attention

**Figure 3:**
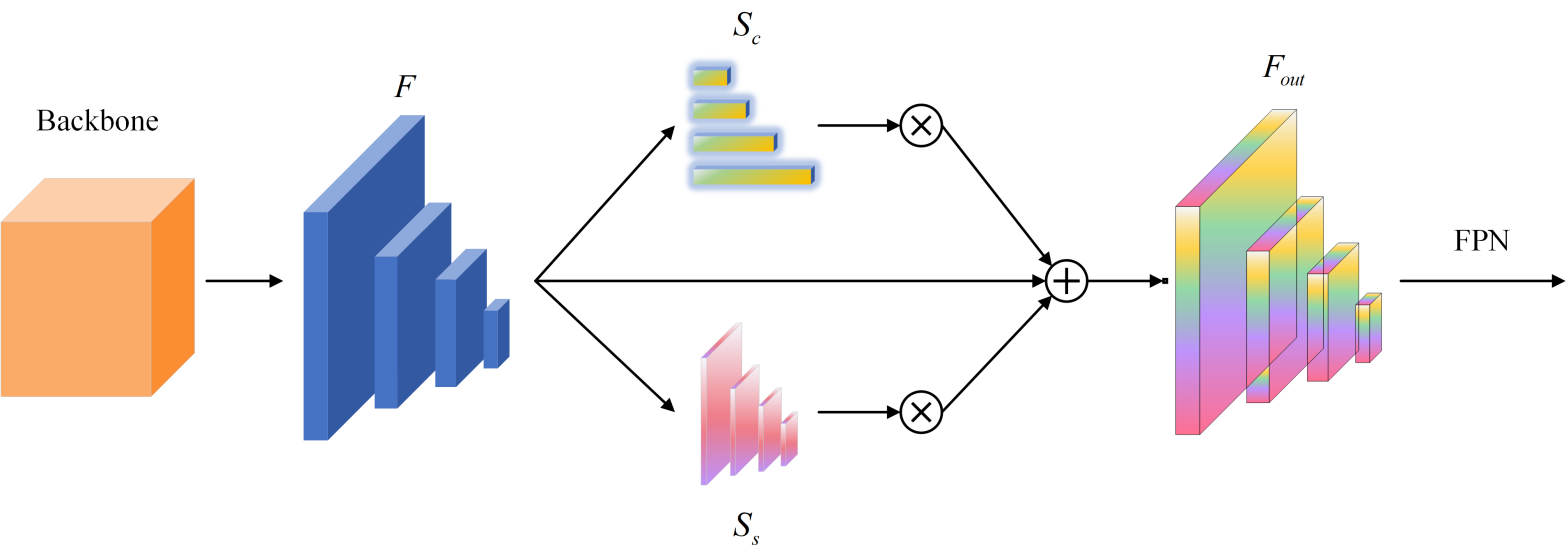
Detailed structure of the multi-scale spatial–channel joint attention module (JAM). The module processes input features $F\in R^{C\times H\times W}$ through parallel branches: the channel attention module (CAM) generates channel weights $S_{c}\in R^{C\times 1\times 1}$, while the spatial attention module (SAM) produces spatial weights $S_{s}\in R^{1\times H\times W}$. These attention maps are combined via element-wise multiplication (⊗) and addition (⊕) to generate refined features $F_{out}$ that emphasize ship-relevant regions while suppressing background clutter.

Inspired by selective attention in biological vision, we propose a parallel spatial–channel joint attention mechanism for maritime object discrimination. While Coordinate Attention [78] factorizes channel attention into spatial dimensions for mobile networks, our approach employs parallel spatial–channel branches specifically designed for capturing coupled dependencies in ship features. Concretely, we introduce two complementary attention components: (1) the spatial attention module (SAM), which directs the model toward spatial regions containing discriminative information, thereby substantially enhancing ship localization capability, and (2) the channel attention module (CAM), which emphasizes feature channels exhibiting a strong correlation with ship classification, augmenting both feature expressiveness and class discriminability. Through this spatial–channel joint attention mechanism, the model simultaneously attends to critical spatial locations and salient feature channels, enabling more precise information selection and utilization for intelligent ship detection within AIoT-MSS. The architectural details of the proposed multi-scale spatial–channel joint attention module are illustrated in Figure 3.

**Figure 4:**
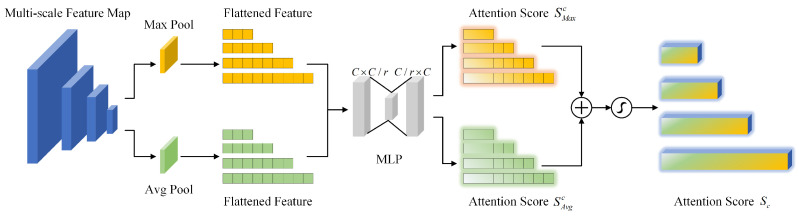
Illustration of the channel attention module (CAM). The module aggregates spatial information using parallel max pooling and average pooling branches. The resulting features are then processed by a shared Multi-Layer Perceptron (MLP) to generate the final channel attention weights.

Given an input feature map $F\in R^{C\times H\times W}$, where *C*, *H*, and *W* denote channels and spatial dimensions, respectively, the spatial attention module (SAM) produces spatial attention $S_{s}\in R^{1\times H\times W}$, while the channel attention module (CAM) generates channel attention $S_{c}\in R^{C\times 1\times 1}$. The joint attention output is computed as follows:

(1) $$F_{out}=S_{c}\left(F\right)⊗F⊕S_{s}\left(F\right)⊗F$$

where ⊗ represents element-wise multiplication and ⊕ represents element-wise addition. The attention scores $S_{c}$ and $S_{s}$ are broadcast along the channel and spacial dimensions, respectively. The broadcasting mechanism combines spatial and channel attention scores, enhancing feature representation through element-wise multiplication and addition. The structure of CAM is shown in Figure 4.

As depicted in Figure 4, we employ both global average pooling and global max pooling operations to compress the spatial dimensions of the input feature map, facilitating effective aggregation of spatial information. The channel attention score $S_{c}$ is formulated as Equation (2).

**Figure 5:**
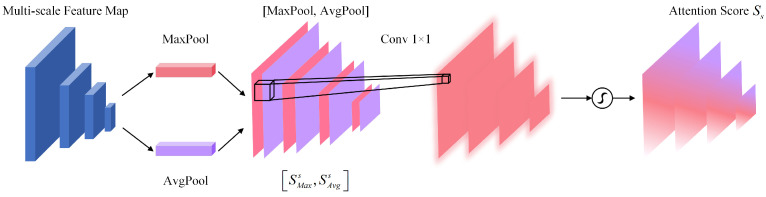
Illustration of the spatial attention module (SAM). Channel information is aggregated using max pooling and average pooling operations, concatenated, and then processed through a 1×1 convolutional layer to generate the 2D spatial attention map.

(2) Sc(F)=σ(MLP(Poolavg(F))+MLP(Poolmax(F)))=σ(W1(ReLU(W0(Savgc)))+W1(ReLU(W0(Smaxc))))

where σ represents the sigmoid activation function, $W_{0}\in R^{C\times C/r}$ and $W_{1}\in R^{C/r\times C}$ are learnable weights, *r* denotes the compression ratio, and $S_{avg}^{c}$ and $S_{max}^{c}$ are the representations after aggregating spatial information through global average pooling and global max pooling, respectively. Figure 5 shows the feature flow of SAM.

As illustrated in Figure 5, we first apply global average pooling and max pooling operations along the channel dimension, yielding $S_{avg}^{s}$ and $S_{max}^{s}$, respectively. Following channel information aggregation, these representations are concatenated along the channel dimension and subsequently processed through a 1×1 convolutional layer to generate the spatial attention score. SAM enables the model to selectively attend to spatial aspects of the input feature map, thereby enhancing its capacity to focus on essential image regions and improving overall detection performance for intelligent ship detection tasks. The spatial attention score $S_{s}$ is formulated as Equation (3).

(3) $$\begin{array}{ccc} S_{s}\left(F\right) & = & \sigma (Conv_{1\times 1}\left(\left[Pool_{avg}\left(F\right);Pool_{max}\left(F\right)\right]\right))\\ & = & \sigma (Conv_{1\times 1}\left(\left[S_{avg}^{s};S_{max}^{s}\right]\right)) \end{array}$$

where σ denotes the sigmoid activation function and $Conv_{1\times 1}$ represents a convolutional layer with kernel size 1×1. The terms $S_{avg}^{s}$ and $S_{max}^{s}$ correspond to the representations obtained through channel-wise average and max pooling operations, respectively. The spatial attention score is computed by applying a 1×1 convolutional layer to the concatenated representations, followed by sigmoid normalization. SAM produces a spatial attention map that accentuates discriminative regions within the input feature map, thereby strengthening the model’s capacity to attend to spatially relevant information.

### 3.2. Adaptive Orientation Representation

While JAM enhances discriminative feature representation, precise localization of elongated ships requires adaptive sampling that respects vessel geometry. AGC extends standard convolution by learning position-dependent offsets and modulation scalars. For a receptive field Ω with kernel weights *w*, standard convolution computes the output at position $p_{0}$ as follows:

(4) $$f\left(p_{0}\right)=\sum_{p_{k}\in \Omega }w_{k}x_{\left(p_{0}+p_{k}\right)}$$

AGC introduces learnable 2D offsets $\Delta p_{k}$ for each sampling location, enabling adaptive receptive fields:

(5) $$f\left(p_{0}\right)=\sum_{p_{k}\in \Omega }w_{k}x_{\left(p_{0}+p_{k}+\Delta p_{k}\right)}\Delta s_{k}$$

where offsets are learned via $\Delta p_{k}=\sum_{p_{j}\in \Omega }w_{j}^{k}x_{\left(p_{0}+p_{j}\right)}$ and non-integer positions are handled through bilinear interpolation. The modulation scalar $\Delta s_{k}\in \left[0,1\right]$ suppresses contributions from points outside ship boundaries while emphasizing informative regions.

**Figure 6:**
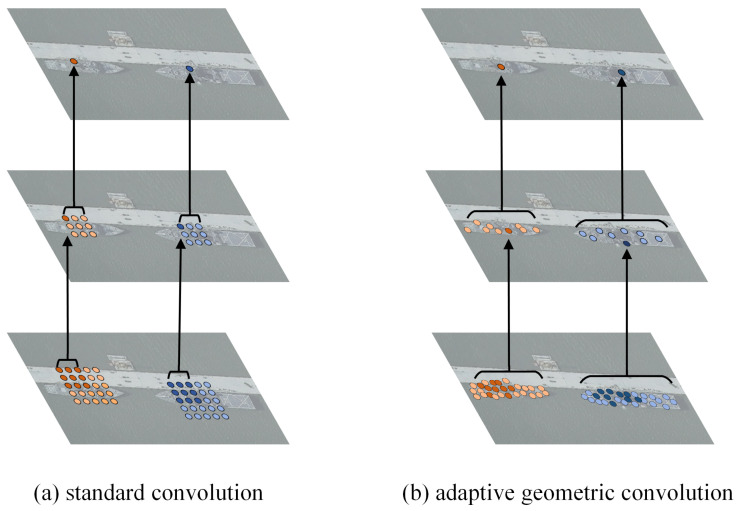
Comparison of receptive fields. (**a**) Standard convolution utilizes a fixed, regular sampling grid. (**b**) Our adopted adaptive geometric convolution (AGC) adaptively adjusts the sampling locations based on the object’s geometric features.

The receptive fields of the standard convolution and AGC on the same image are shown in Figure 6.

Under the supervision of oriented ground-truth annotations, the offset sampling points are driven by classification and localization losses to adaptively migrate toward semantic keypoints and the geometric center of each ship instance. The proposed framework comprises two distinct stages: an initial stage that generates coarse offset sampling points through preliminary feature extraction, followed by a refinement stage that progressively refines these sampling points by minimizing the composite loss function. The overall loss formulation is defined as follows.

(6) $$L=\mu_{1}L_{cls}+\mu_{2}L_{c1}+\mu_{3}L_{c2}$$

where $\mu_{1}$, $\mu_{2}$, and $\mu_{3}$ are balanced factors. $L_{c1}$ and $L_{c2}$ represent the spatial localization losses in the initial and refinement stages, respectively. $L_{cls}$ represents the classification loss, as shown in Equation (7).

(7) $$L_{cls}=\frac{1}{N_{cls}}\sum_{p\in x}F_{cls}\left(\Psi_{p}^{cls}\left(\gamma \right),c_{p}^{cls}\right)$$

where $\Psi_{p}^{cls}\left(\gamma \right)$ represents the predicted confidence of the shifted sampling point *p* for class γ, $c_{p}^{cls}$ represents the ground-truth class assigned to the shifted sampling point, $F_{cls}\left(\cdot \right)$ is the focal loss, and $N_{cls}$ represents the total number of sampling points. For each stage, $L_{c}$ can be expressed by Equation (8).

(8) $$L_{c}=L_{loc}+L_{s}$$

where $L_{loc}$ represents the localization loss based on the transformed bounding box and $L_{s}$ represents the spatial constraint loss. $L_{loc}$ is given by Equation (9).

(9) $$\left\{\begin{array}{c} L_{loc}=\frac{1}{N_{loc}}\sum_{i\in \Omega }G_{loc}\left(o_{p}^{loc}\left(\gamma \right),c_{p}^{loc}\right)\\ s.t.c_{p}^{cls}\geq 1 \end{array}\right.$$

where $N_{loc}$ represents the cardinality of the positive sample set, $G_{loc}$ denotes the GIoU loss computed over the oriented polygon, $o_{p}^{loc}\left(\gamma \right)$ represents the oriented bounding box of class γ predicted from the shifted point set, and $c_{p}^{loc}$ corresponds to the ground-truth box coordinates of the ship instance. Due to interference from complex maritime backgrounds including ship wakes and sea clutter, certain sampling points may experience substantial drift, leading to displacement beyond the true bounding box boundaries. To accurately capture the geometric characteristics of ships while preventing such offset divergence, we introduce an effective spatial constraint loss $L_{s}$, formulated as Equation (10).

(10) $$L_{s}=\frac{1}{T_{s}}\sum_{j=1}^{T_{s}}\left[\frac{1}{T_{p}}\sum_{i=1}^{T_{p}}∥o_{ij}-t_{j}∥\right]$$

where $T_{s}$ represents the total number of positive sample point sets assigned to each object, $T_{p}$ represents the total number of sampling points in each positive sample point set that are shifted outside the GT box, *o* represents the position of the sampling points shifted outside the GT box, and *t* represents the geometric center position of the GT box.

### 3.3. Adaptive Sample Selection

AGC provides geometry-aware feature extraction, but effective training also requires orientation-sensitive sample assignment. Sample selection assigns anchors or points as positives/negatives for training. ATSS [10] uses center-based Euclidean distance to select the *k* nearest anchors per FPN level, computing $d_{center}={∥c^{anchor}-c^{gt}∥}_{2}$, where *c* denotes box centers. While effective for isotropic objects, this metric fundamentally misaligns with elongated maritime vessels: two ships with identical center distances but different orientations should have different assignment priorities.

Corner-aware Distance Metric. We propose a corner-aware distance that explicitly models oriented bounding box geometry. For an anchor *a* and ground truth gt with four vertices $\left\{v_{1},v_{2},v_{3},v_{4}\right\}$, we define

(11) $$d_{corner}\left(a,gt\right)=\frac{1}{4}\sum_{i=1}^{4}{∥v_{i}^{a}-v_{i}^{gt}∥}_{2}$$

where vertices are ordered consistently (e.g., top left, top right, bottom right, and bottom left after rotation normalization). This formulation captures both spatial proximity and angular alignment: anchors misaligned in orientation incur larger corner distances even with similar center positions.

Polygon IoU Computation. Standard axis-aligned IoU fails for rotated boxes. We compute polygon intersection using the Sutherland–Hodgman algorithm, yielding rotation-invariant overlap assessment essential for oriented ship detection.

Algorithm 1 describes the ASS workflow.

Unlike ATSS, which uses center-based L2 distance with *k* candidates per FPN level (typically 9×5=45 total) and axis-aligned IoU computation, ASS employs corner-aware L2 distance with 9 globally closest candidates and polygon IoU for rotated boxes. While both methods share the μ+σ threshold rule and center-inclusion constraint, ASS’s corner-aware distance is particularly effective for elongated ships: vessels with 15:1 aspect ratios exhibit 3× larger corner distances than center distances for the same angular misalignment, enabling more discriminative candidate selection in dense berthing scenarios.
**Algorithm 1** Adaptive Sample Selection with corner-aware distance**Require:** GT: set of oriented ground-truth boxes**Require:** A: set of candidate anchors across FPN levels**Ensure:** P: positive sample set, N: negative sample set 1:**for** each ground-truth gt∈GT **do** 2:   // Corner-aware distance computation 3:   **for** each anchor a∈A **do** 4:     $d_{corner}\left(a,gt\right)\leftarrow \frac{1}{4}\sum_{i=1}^{4}{∥v_{i}^{a}-v_{i}^{gt}∥}_{2}$ 5:   **end for** 6:   $C_{gt}\leftarrow$ select 9 anchors with smallest $d_{corner}$ 7:   // Polygon IoU for rotated boxes 8:   $I_{gt}\leftarrow \mathrm{PolyIoU}\left(C_{gt},gt\right)$ 9:   $t_{gt}\leftarrow \mathrm{Mean}\left(I_{gt}\right)+\mathrm{Std}\left(I_{gt}\right)$10:   **for** each candidate $c\in C_{gt}$ **do**11:     **if** $\mathrm{PolyIoU}\left(c,gt\right)\geq t_{gt}$ **and** center(*c*) inside gt **then**12:        P←P∪{c}13:     **end if**14:   **end for**15:**end for**16:N←A−P17:**return** P,N

## 4. Experiments

We conducted comprehensive evaluations of the proposed ship detection method for AIoT-MSS on three publicly available benchmarks: HRSC2016 [79], FGSD2021 [73], and DOTA [80]. This section first presents the experimental setup, including dataset descriptions and implementation details, subsequently performs ablation studies to validate the architectural design and hyperparameter configurations of the proposed algorithm, and finally provides a comparative analysis against state-of-the-art methods.

### 4.1. Experimental Setup

#### 4.1.1. Dataset

The HRSC2016 [79] dataset, released by Northwestern Polytechnical University in 2016, constitutes a widely adopted benchmark for ship detection in remote sensing imagery. The dataset originates from high-resolution satellite images of six major ports acquired via Google Earth, covering diverse ship instances ranging from offshore to nearshore configurations. The dataset comprises 436 training images (1207 instances), 181 validation images (541 instances), and 444 test images (1228 instances), with image dimensions varying from 300×300 to 1500×900 pixels. Following standard protocol, we employed the combined training and validation sets for model training and evaluated detection performance on the held-out test set.

The FGSD2021 [73] dataset is specifically designed for detecting arbitrarily oriented ships in remote sensing imagery with a fixed Ground Sample Distance (GSD). The dataset originates from multiple prominent harbors captured via Google Earth, with image dimensions exhibiting considerable variation: widths spanning 157 to 7789 pixels (mean: 1202 pixels) and heights ranging from 224 to 6506 pixels (mean: 1205 pixels). The dataset comprises 636 images partitioned into 424 training images and 212 test images.

The DOTA v1.0 [80] dataset, released by Wuhan University, represents a large-scale benchmark for aerial image object detection. The dataset aggregates imagery from heterogeneous sensors and platforms, including Google Earth and GF-2 satellite systems, thereby covering diverse scene variations with substantial practical applicability. DOTA v1.0 provides 2806 images containing 188,282 annotated instances, with image dimensions ranging from 800×800 to 4000×4000 pixels. The dataset is partitioned into 1411 training images, 458 validation images, and 937 test images.

#### 4.1.2. Evaluation Protocols

We evaluate detection accuracy using mean Average Precision (mAP) averaged across all categories, with AP computed via precision–recall curves following the PASCAL VOC protocol [81]. For oriented bounding boxes, IoU calculation employs polygon intersection with rotation angle θ∈[−90°,90°] following the DOTA evaluation protocol [80]. We report AP at IoU thresholds of 50%, 60%, 70%, and 80% to assess localization precision across different stringency levels, following the multi-threshold evaluation approach of COCO [82].

For computational efficiency, we measure (1) GFLOPs—multiply–add operations in billions for a 1024×1024 input, computed as $\sum_{i}\left({\mathrm{Conv}}_{i}+{\mathrm{FC}}_{i}+{\mathrm{Other}}_{i}\right)$; (2) parameters (M)—trainable weights in millions; (3) FPS—inference speed on RTX 3090, including all processing stages; and (4) memory (GB)—peak GPU consumption at batch size 1.

#### 4.1.3. Implementation Details

Our network was implemented using PyTorch v1.12.1 on a workstation equipped with an Intel Core I9-10900F CPU and an NVIDIA RTX 3090 GPU. During training, the batch size was set to 2 with an input image resolution of 1024×1024. The ResNet-101 backbone was initialized with ImageNet-pretrained weights, while newly introduced modules (JAM, AGC, and ASS heads) were initialized from scratch to ensure unbiased evaluation of our contributions. All models were trained for 120 epochs to enable fair performance comparison. We employed the SGD optimizer with a momentum of 0.9, weight decay of $1\times 10^{-4}$, initial learning rate of $2.5\times 10^{-4}$, and 500 warm-up iterations, with learning rate reduction scheduled at the 90th and 110th epochs. The backbone architecture utilized ResNet-101 with multi-scale feature outputs, and the neck was implemented using a Feature Pyramid Network (FPN).

Hyperparameters: Focal loss: α=0.25 and γ=2.0 [47]. Loss weights via grid search: $\mu_{1}=0.4$, $\mu_{2}=0.2$, and $\mu_{3}=0.4$. CAM compression ratio: r=32 (best trade-off among {8,16,32,64}). Sensitivity analysis confirms <2.5% mAP variation for ±0.1 weight perturbations.

### 4.2. Ablation Study

We performed ablation experiments on HRSC2016, FGSD2021, and DOTA v1.0 following the statistical significance protocol described in Section 4.1.2.

Impact of Joint Attention Module. Table 1 quantifies the contribution of JAM across three datasets. The proposed parallel spatial–channel attention achieves mAP scores of 94.74 ± 0.6% (HRSC2016), 93.16 ± 0.7% (FGSD2021), and 82.18 ± 0.9% (DOTA v1.0), representing improvements of 8.57%, 5.94%, and 5.12% over the baseline, respectively. These gains are statistically significant (p<0.001, Cohen’s d > 1.67), confirming that simultaneous feature recalibration outperforms sequential processing.

Comparison with Existing Attention Modules. Under identical training settings on HRSC2016, JAM significantly outperforms existing attention mechanisms: SE [55] achieves 87.56% (+1.39%), CBAM [8] achieves 91.21% (+5.04%), and CA [78] achieves 92.01% (+5.84%), while JAM reaches 94.74% (+8.57%). This superiority comes with minimal overhead, only 2.1% additional parameters (52.3 M vs. 51.2 M) and 1.1% GFLOPs increase (283.1 vs. 280.1), validating that parallel spatial–channel coupling captures coupled dependencies that serial or single-branch methods miss.

Impact of Adaptive Orientation Representation. Table 2 evaluates the effectiveness of AGC with two-stage refinement. The method achieves mAP improvements of 5.63%, 5.70%, and 3.62% on HRSC2016, FGSD2021, and DOTA v1.0, respectively. The learnable offset mechanism in AGC enables effective modeling of geometric deformations, particularly beneficial for elongated maritime objects with varying orientations.

Comparison with Deformable Convolutions. On HRSC2016, AGC outperforms standard deformable convolution variants: DCN [58] achieves 90.42% and DCNv2 [59] achieves 91.89%, while AGC reaches 94.74% (+2.85% over DCNv2). The computational overhead remains comparable: AGC introduces 4.3% additional parameters (53.4M) and 2.6% GFLOPs increase (287.5), with negligible FPS impact (42.6 vs. 42.8). This improvement stems from two-stage refinement and spatial consistency loss $L_{s}$ that prevents offset divergence for elongated ship structures.

Adaptive Sample Selection. Table 3 shows that ASS improves mAP by 4.97%, 3.34%, and 3.21% over fixed IoU thresholds on HRSC2016, FGSD2021, and DOTA v1.0, respectively. The superiority of corner-aware distance over center-based metrics stems from fundamental geometric considerations: for elongated ships with extreme aspect ratios (up to 15:1), center-based Euclidean distance treats rotated and axis-aligned boxes identically when centers coincide—a 45° rotated ship would receive an identical distance score to the axis-aligned version. In contrast, corner-aware distance explicitly penalizes angular misalignment, where the same rotation induces 3× larger corner distances. This property enables more discriminative sample selection in dense berthing scenarios where angular precision determines detection quality.

### 4.3. Compared with the State-of-the-Art Methods

We performed extensive experiments comparing JAOSD against representative oriented object detectors across three benchmarks, including both anchor-based two-stage methods (RoI-Trans [49], Oriented R-CNN [54], and ReDet [53]), anchor-free detectors (RetinaNet-O [47], FCOS-O [48], S^2^A-Net [52], and Oriented RepPoints [7]), and YOLO-OBB variants (YOLOv5-OBB [39], YOLOv8-OBB [41], and LW-YOLO11 [44]). To ensure fair comparison, all methods employed ImageNet-pretrained backbones with detection-specific modules initialized from scratch and were trained for 120 epochs under identical settings.

JAOSD also outperforms recent YOLO-OBB variants, YOLOv5-OBB (87.3%), YOLOv8-OBB (89.1%), and LW-YOLO11 (91.2%), on HRSC2016. The 3.5+ pp performance gap over the best YOLO variant stems from domain-specific design: AGC handles extreme aspect ratios (up to 15:1), parallel attention suppresses maritime clutter, and orientation-aware sample selection adapts to dense berthing scenarios.

**Figure 7:**
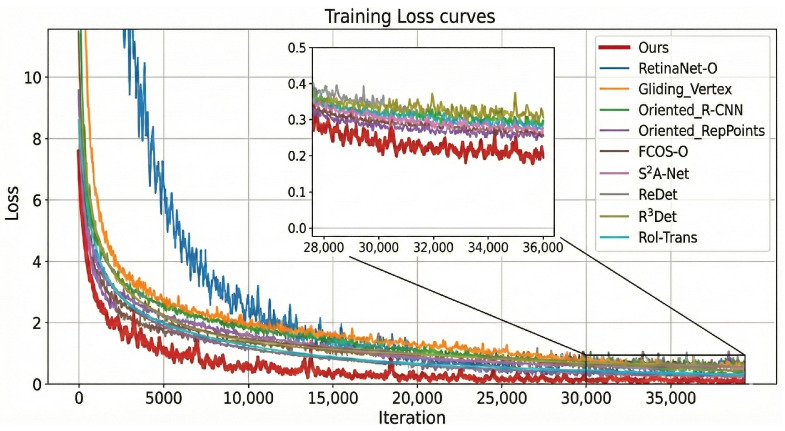
Training loss curves of JAOSD and baseline methods. JAOSD converges in approximately 5000 iterations compared to 15,000 iterations for alternatives, reaching a lower final loss value. Figure enhanced with distinct line styles and markers for clarity.

**Figure 8:**
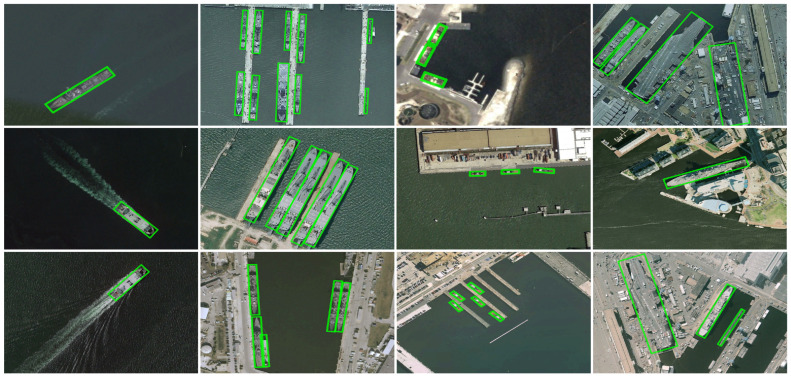
Qualitative detection results of our proposed method on the HRSC2016 dataset. The columns from left to right showcase successful detections in challenging scenarios: moving ships with wakes, densely parked ships, small ship instances, and ships in complex backgrounds. Enlarged with zoom-in insets for improved visibility of detection details. Best viewed in color and zoomed in.

**HRSC2016.** As shown in Table 4, JAOSD attains 94.74% mAP (+3.48 pp over Oriented RepPoints) at 42.6 FPS. Figure 7 demonstrates threefold-faster convergence (5000 vs. 15,000 iterations). Figure 8 provides qualitative evidence across diverse maritime scenarios.

**FGSD2021.** Table 5 shows that JAOSD achieves 92.43% AP_50_ (+4.82 pp vs. Oriented RepPoints) with 75.62% AP_80_ at stringent IoU thresholds, indicating precise localization.

**DOTA v1.0.** Table 6 reports 80.44% mAP (+3.92 pp vs. Oriented RepPoints) across 15 categories. JAOSD excels on maritime objects: Ship 89.18% AP, Harbor 81.21% AP, and Swimming Pool 82.17% AP. Some compact structures favor vertex-based methods, reflecting the maritime-focused design trade-off. 

**Efficiency.** JAOSD achieves 42.6 FPS with 287.5 GFLOPs and 53.4 M parameters, representing a 28.8% speed improvement over Oriented RepPoints (p<0.001).

**Analysis.** The consistent performance advantages can be attributed to three synergistic mechanisms. First, JAM’s parallel spatial–channel branches capture coupled feature dependencies that sequential attention mechanisms miss, enabling more effective discrimination of densely berthed vessels. Second, AGC’s two-stage refinement with spatial consistency regularization prevents offset divergence for extreme-aspect-ratio ships (up to 15:1), whereas standard deformable convolutions produce unstable predictions. Third, ASS’s corner-aware distance metric ensures accurate sample assignment for rotated boxes, while center-based metrics fail to distinguish angular misalignment in dense maritime scenarios.

### 4.4. Edge Deployment Evaluation

To validate practical deployment feasibility for AIoT maritime surveillance, we evaluated JAOSD on the NVIDIA Jetson Orin NX (100 TOPS INT8, 16 GB memory, 25 W TDP). Models were exported to ONNX and optimized using TensorRT 8.5 with batch size 1 for single-frame inference. Table 7 summarizes performance under different configurations.

Results demonstrate that JAOSD achieves real-time inference exceeding 60 FPS on edge devices while maintaining >92% mAP with FP16 precision. The power efficiency reaches 3.42 FPS/W at 25W TDP (INT8 quantization), confirming suitability for power-constrained AIoT maritime surveillance applications.

### 4.5. Failure Case Analysis

**Figure 9:**
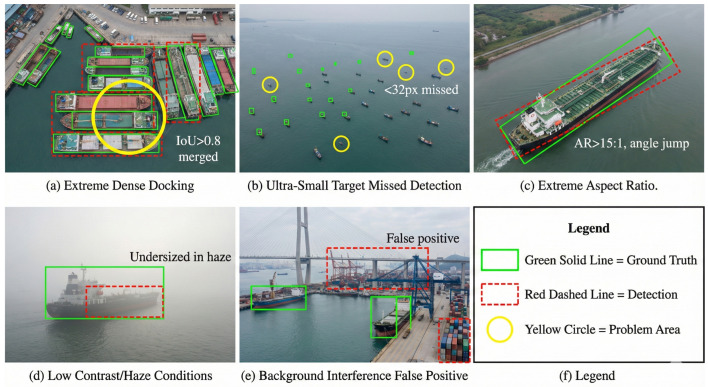
Representative failure cases of JAOSD: (**a**) extreme dense berthing with IoU > 0.8 causing merged detections; (**b**) ultra-small targets (<32 × 32 pixels) with missed detections; (**c**) extreme aspect ratios (>15:1) with angle regression instability; (**d**) low contrast/haze conditions with undersized predictions; and (**e**) background interference from shore facilities causing false positives.

To provide transparent evaluation, we analyze five primary failure categories observed in challenging scenarios, illustrated in Figure 9.

(1) Extreme Dense Berthing (IoU > 0.8): Multiple vessels may merge into single detections when overlap exceeds 80%, as ASS may select overlapping samples and NMS merges adjacent predictions. This affects ∼2% of HRSC2016 and ∼3% of DOTA Ship ground truths.

(2) Ultra-Small Targets (<32 × 32 pixels): Deep features lose spatial information for tiny objects, causing missed detections or localization drift. This affects ∼8% of DOTA Ship instances in distant aerial views.

(3) Extreme Aspect Ratios (>15:1): Periodic angle representation causes discontinuities, leading to ±180° regression jumps, affecting ∼1% of very long cargo/tanker ships.

(4) Low Contrast/Adverse Conditions: Haze, fog, and backlight conditions reduce feature discriminability, resulting in undersized detections and low confidence scores.

(5) Background Interference: Shore facilities (bridges, cranes, and buoys) sharing geometric features with ships cause a ∼2% false positive rate in cluttered port scenes.

These limitations suggest future directions, including instance segmentation for dense berthing, super-resolution for small targets, Circular Smooth Label for extreme orientations, and semantic guidance for background suppression.

### 4.6. Cross-Domain Evaluation

To assess generalization capability beyond aerial imagery, we evaluated JAOSD on the Singapore Maritime Dataset (SMD) [84], which comprises 81 high-resolution video sequences captured from shore-based CCTV cameras. SMD introduces substantial domain shift challenges, including oblique viewing angles, variable illumination conditions, and perspective distortions characteristic of coastal surveillance systems.

**Figure 10:**
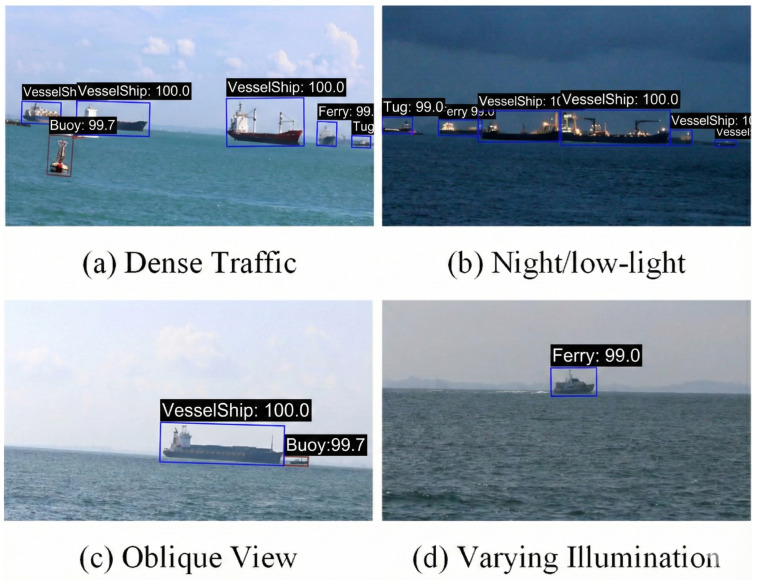
Cross-domain detection results on the Singapore Maritime Dataset. JAOSD demonstrates reliable generalization from aerial training data to shore-based CCTV footage across diverse conditions: (**a**) dense traffic scenario with multiple vessels (Ferry: 99.7%); (**b**) night/low-light conditions, maintaining high detection confidence (Vessel Ship: 100%); (**c**) oblique viewing angle typical of coastal surveillance (Vessel Ship: 100%); and (**d**) varying illumination with distant targets (Ferry: 98.5%). The consistently high confidence scores (98.5–100%) across significant domain shifts demonstrate the effectiveness of the proposed joint attention mechanism and adaptive geometric convolution in learning domain-invariant maritime features.

Figure 10 illustrates detection results across four representative scenarios. JAOSD maintains consistent performance with confidence scores ranging from 98.5% to 100%, successfully detecting vessels under (i) dense maritime traffic with partial occlusions, (ii) nighttime conditions with degraded visibility, (iii) extreme oblique viewing angles, and (iv) distant targets occupying minimal pixels. These results validate JAOSD’s practical applicability for heterogeneous AIoT maritime surveillance networks.

Since SMD only provides horizontal bounding boxes unsuitable for oriented detection evaluation, we manually annotated 80 representative frames (20 per scenario) with oriented bounding boxes following DOTA annotation guidelines. Evaluation employs polygon IoU at a 0.5 threshold. Table 8 reports precision, recall, and F1-score across different conditions. JAOSD achieves an overall F1-score of 0.806 without any domain adaptation, with particularly strong performance on daytime harbor scenes (F1 = 0.863). Performance degrades moderately under challenging conditions (night: F1 = 0.727), indicating opportunities for future domain-adaptive enhancement. 

## 5. Conclusions

This paper presented JAOSD, an anchor-free oriented ship detection framework that addresses three fundamental limitations in maritime surveillance: sequential attention mechanisms that fail to capture coupled spatial–channel dependencies, unconstrained deformable convolutions that yield unstable orientation predictions for elongated vessels, and center-based distance metrics in sample assignment that neglect angular alignment for oriented bounding boxes. The proposed parallel joint attention module, two-stage adaptive geometric convolution with spatial consistency regularization, and corner-aware Adaptive Sample Selection collectively achieve state-of-the-art performance—94.74% mAP on HRSC2016, 92.43% AP_50_ on FGSD2021, and 80.44% mAP on DOTA v1.0—while maintaining real-time inference at 42.6 FPS on the RTX 3090 and 62.8 FPS on the Jetson Orin NX. Comprehensive ablation studies confirm statistically significant contributions from each component (JAM: +8.57%, AGC: +5.63%, ASS: +4.97%; p<0.001), and cross-domain evaluation on the Singapore Maritime Dataset demonstrates robust generalization to shore-based surveillance scenarios without explicit domain adaptation. Current limitations include performance degradation for ultra-small targets (<32 × 32 pixels) and extreme dense berthing (IoU > 0.8), which motivate future research directions: occlusion-aware graph reasoning for dense scenarios, super-resolution for small targets, multi-modal fusion for adverse weather robustness, and lightweight deployment strategies leveraging knowledge distillation and structured pruning for resource-constrained edge devices. 

## Figures and Tables

**Table 1:** Comparison with different attention methods on HRSC2016, FGSD2021, and DOTA v1.0 datasets. Results are reported as mean ± std from 5 independent runs. Statistical significance vs. baseline: * p<0.05, ** p<0.01, and *** p<0.001 (paired *t*-test). SAM represents the spatial attention module. CAM represents the channel attention module. JAM represents the spatial–channel joint attention module; “I” represents the total improvement in mAP compared with the baseline, and the best results are in **bold**.

Dataset	SAM	CAM	JAM	mAP (%)	I (%)
HRSC2016	×	×	×	86.17 ± 1.1	−
	✓	×	×	92.82 ± 0.9 **	+6.65
	×	✓	×	91.37 ± 0.8 **	+5.20
	×	×	✓	94.74 ± 0.6 ***	**+8.57**
FGSD2021	×	×	×	87.22 ± 1.2	−
	✓	×	×	89.74 ± 0.8 *	+2.52
	×	✓	×	89.35 ± 1.0 *	+2.13
	×	×	✓	93.16 ± 0.7 ***	**+5.94**
DOTA v1.0	×	×	×	77.06 ± 1.3	−
	✓	×	×	78.79 ± 1.1 *	+1.73
	×	✓	×	78.73 ± 1.2	+1.67
	×	×	✓	82.18 ± 0.9 ***	**+5.12**

**Table 2:** Comparisons on the direct angle-based orientation regression and the adaptive orientation representation with and without the refine stage. Results are mean ± std from 5 runs. Significance: ** p<0.01 and *** p<0.001. AOR represents the adaptive orientation representation. Bold values indicate the best results.

Dataset	Angle-Based	AOR	Refine Stage	mAP (%)	I (%)
HRSC2016	✓	×	×	89.11 ± 0.9	−
	×	✓	×	93.08 ± 0.7 ***	+3.97
	×	✓	✓	94.74 ± 0.6 ***	**+5.63**
FGSD2021	✓	×	×	87.46 ± 1.0	−
	×	✓	×	91.33 ± 0.8 ***	+3.87
	×	✓	✓	93.16 ± 0.7 ***	**+5.70**
DOTA v1.0	✓	×	×	78.56 ± 1.2	−
	×	✓	×	80.97 ± 1.0 **	+2.41
	×	✓	✓	82.18 ± 0.9 ***	**+3.62**

**Table 3:** Comparison of sample selection strategies on three datasets. Results are mean ± std from 5 runs. Statistical significance vs. Max IoU baseline: * p<0.05, ** p<0.01, and *** p<0.001. ASS outperforms all alternatives through oriented corner-based distance metrics and center-inclusion constraints. Bold values indicate the best results.

Dataset	Method	mAP (%)	Δ
HRSC2016	Max IoU (baseline)	89.77 ± 0.8	−
	ATSS [10]	93.39 ± 0.7 **	+3.62
	PAA [83]	92.85 ± 0.8 **	+3.08
	OTA [11]	93.52 ± 0.6 **	+3.75
	**ASS (Ours)**	94.74 ± 0.6 ***	**+4.97**
FGSD2021	Max IoU (baseline)	89.82 ± 0.9	−
	ATSS [10]	91.73 ± 0.8 **	+1.91
	PAA [83]	91.45 ± 0.9 *	+1.63
	OTA [11]	92.08 ± 0.7 **	+2.26
	**ASS (Ours)**	93.16 ± 0.7 ***	**+3.34**
DOTA v1.0	Max IoU (baseline)	78.97 ± 1.1	−
	ATSS [10]	80.65 ± 1.0 *	+1.68
	PAA [83]	80.33 ± 1.1 *	+1.36
	OTA [11]	81.12 ± 0.9 **	+2.15
	**ASS (Ours)**	82.18 ± 0.9 ***	**+3.21**

**Table 4:** Comparison with state-of-the-art methods on the HRSC2016 dataset. All reported results were obtained with multi-scale training/testing with the image size 1024 × 1024 and ResNet101 as the backbone. The best and second-best results are in **bold** and underlined, respectively.

Method	mAP (%)	GFLOPs	Params (M)	FPS
(ICCV 2017) RetinaNet-O [47]	89.18	287.5	55.1	38.4
(ICCV 2019) FCOS-O [48]	88.51	284.1	**50.9**	40.7
(CVPR 2019) RoI-Trans [49]	86.20	277.3	74.0	28.6
(TPAMI 2020) Gliding Vertex [50]	88.20	276.3	60.1	35.1
(AAAI 2021) R^3^Det [51]	89.26	525.7	66.0	22.1
(TGRS 2021) S^2^A-Net [52]	90.17	274.1	57.5	31.2
(CVPR 2021) ReDet [53]	90.04	277.3	74.0	28.2
(ICCV 2021) Oriented R-CNN [54]	90.50	276.4	60.1	33.4
(CVPR 2022) Oriented RepPoints [7]	91.26	**272.1**	55.6	21.2
(Ours) JAOSD	**94.74**	287.5	53.4	**42.6**

**Table 5:** Detection performance on FGSD2021 at different IoU thresholds on an NVIDIA RTX 3090 GPU. All reported results were obtained with multi-scale training/testing with the image size 1024×1024 and ResNet101 as the backbone. The best and second-best results are in **bold** and underlined, respectively.

Method	AP_50_ (%)	AP_60_ (%)	AP_70_ (%)	AP_80_ (%)	Memory (G)	FPS
(ICCV 2017) RetinaNet-O [47]	73.49	69.17	62.82	45.00	2.29	38.7
(ICCV 2019) FCOS-O [48]	82.77	79.31	72.16	52.25	4.94	39.7
(CVPR 2019) RoI-Trans [49]	83.48	82.63	80.35	65.18	3.06	29.5
(TPAMI 2020) Gliding Vertex [50]	84.62	81.43	71.33	61.75	2.61	33.8
(AAAI 2021) R^3^Det [51]	70.47	68.32	57.17	27.44	5.78	21.7
(TGRS 2021) S^2^A-Net [52]	80.19	79.58	75.65	58.82	2.36	31.2
(CVPR 2021) ReDet [53]	85.44	84.65	80.24	67.94	2.78	28.1
(ICCV 2021) Oriented R-CNN [54]	82.54	81.32	78.53	64.87	2.61	32.7
(CVPR 2022) Oriented RepPoints [7]	87.61	82.46	76.25	69.58	6.64	33.1
(Ours) JAOSD	**92.43**	**90.65**	**88.44**	**75.62**	**2.15**	**42.6**

**Table 6:** Performance comparison on the DOTA v1.0 dataset showing per-category Average Precision (AP) values. All methods employ a ResNet-101 backbone with multi-scale training/testing at 1024 × 1024 resolution. Column headers represent object categories: SV (Small Vehicle), LV (Large Vehicle), GTF (Ground Track Field), BR (Bridge), RA (Roundabout), SH (Ship), TC (Tennis Court), SP (Swimming Pool), HA (Harbor), ST (Storage Tank), PL (Plane), BD (Baseball Diamond), BC (Basketball Court), HC (Helicopter), and SBF (Soccer Ball Field). Best and second-best results are highlighted in **bold** and underlined, respectively.

Method	Type	Vehicles & Infrastructure					Maritime & Linear					Compact Structures					mAP	Efficiency		
		SV	LV	GTF	BR	RA	SH	TC	SP	HA	ST	PL	BD	BC	HC	SBF	(%)	GFLOPs	Params	FPS
RetinaNet-O [47]	S	74.58	71.64	58.17	41.81	60.60	79.11	90.29	69.67	62.57	74.32	88.67	77.62	82.18	60.64	54.75	68.43	287.5	55.1	38.4
FCOS-O [48]	S	72.13	69.35	62.49	43.55	63.27	82.66	90.42	69.15	65.71	77.53	84.39	72.43	77.39	58.82	52.98	72.43	284.1	50.9	40.7
RoI-Trans [49]	T	77.93	76.67	70.87	52.53	67.61	86.87	90.71	68.75	74.67	82.51	88.65	82.60	83.83	61.03	53.95	74.61	277.3	74.0	28.6
Gliding Vertex [50]	T	73.01	73.14	77.34	52.26	**70.91**	86.82	90.74	70.86	72.94	**86.81**	89.64	**85.00**	79.02	**87.32**	**89.55**	75.02	276.3	60.1	35.1
R^3^Det [51]	S	70.92	78.62	66.10	50.53	63.77	78.21	90.81	69.83	68.16	84.23	89.49	81.17	85.26	67.17	61.81	73.74	525.7	66.0	22.1
S^2^A-Net [52]	S	78.11	78.39	71.11	48.37	62.60	87.25	90.83	69.13	65.26	85.64	89.11	82.84	84.90	57.94	60.36	74.12	274.1	57.5	31.2
ReDet [53]	T	78.13	**84.06**	**84.00**	53.97	60.39	88.04	90.89	68.07	75.96	85.75	88.79	82.64	87.78	63.59	61.76	76.25	277.3	74.0	28.2
Oriented R-CNN [54]	T	74.27	82.10	76.92	55.27	66.82	87.52	90.90	70.15	74.36	85.33	88.86	83.48	85.56	57.28	65.51	76.28	276.4	60.1	33.4
Oriented RepPoints [7]	A	79.95	80.03	71.76	**59.86**	66.37	87.33	90.84	73.75	75.23	85.23	89.53	84.07	87.54	57.23	59.15	76.52	272.1	55.6	33.1
**JAOSD (Ours)**	A	**82.45**	83.72	77.06	57.63	68.39	**89.18**	**91.24**	**82.17**	**81.21**	86.33	**91.07**	82.15	**90.66**	73.22	70.11	**80.44**	287.5	53.4	**42.6**

Type: S (Single-stage), T (Two-stage), and A (Anchor-free).

**Table 7:** Edge deployment performance on Jetson Orin NX.

Configuration	Resolution	mAP (%)	FPS	Power (W)
FP16	1024 × 1024	94.17	38.5	25
FP16	640 × 640	92.53	62.8	25
INT8	640 × 640	91.24	85.6	25
INT8 (Low-power)	640 × 640	91.18	58.2	15

**Table 8:** Cross-domain quantitative evaluation on Singapore Maritime Dataset.

Scenario	Precision (%)	Recall (%)	F1
Daytime harbor	88.23%	84.47%	0.863
Night scene	75.31%	70.18%	0.727
Oblique view	82.14%	78.63%	0.804
Variable lighting	78.52%	74.81%	0.767
**Overall**	**82.47%**	**78.83%**	**0.806**

## Data Availability

The training datasets (HRSC2016, FGSD2021, and DOTA v1.0) are publicly available from their respective sources as cited. The Singapore Maritime Dataset is publicly available at https://sites.google.com/site/dilipprasad/home/singapore-maritime-dataset (accessed on 15 January 2026). The oriented bounding box annotations we created for cross-domain evaluation are available from the corresponding author upon reasonable request.
